# Developing a model of short-term integrated palliative and supportive care for frail older people in community settings: perspectives of older people, carers and other key stakeholders

**DOI:** 10.1093/ageing/afw124

**Published:** 2016-11-02

**Authors:** Anna E. Bone, Myfanwy Morgan, Matthew Maddocks, Katherine E. Sleeman, Juliet Wright, Shamim Taherzadeh, Clare Ellis-Smith, Irene J. Higginson, Catherine J. Evans

**Affiliations:** 1 Department of Palliative Care, Policy and Rehabilitation, Cicely Saunders Institute, King's College London, London, UK; 2 Institute of Pharmaceutical Science, King's College London, London, UK; 3 Department of Geriatric Medicine, Brighton and Sussex Medical School, Brighton, UK; 4 Northbourne Medical Centre, Shoreham-by-Sea, West Sussex, UK; 5 Sussex Community NHS Trust, Brighton and Hove, UK

**Keywords:** frail older people, palliative care, primary health care, qualitative research, consensus

## Abstract

**Background:**

understanding how best to provide palliative care for frail older people with non-malignant conditions is an international priority. We aimed to develop a community-based episodic model of short-term integrated palliative and supportive care (SIPS) based on the views of service users and other key stakeholders in the United Kingdom.

**Method:**

transparent expert consultations with health professionals, voluntary sector and carer representatives including a consensus survey; and focus groups with older people and carers were used to generate recommendations for the SIPS model. Discussions focused on three key components of the model: potential benefit of SIPS, timing of delivery and processes of integrated working between specialist palliative care and generalist practitioners. Content and descriptive analysis was employed and findings were integrated across the data sources.

**Findings:**

we conducted two expert consultations (*n* = 63), a consensus survey (*n* = 42) and three focus groups (*n* = 17). Potential benefits of SIPS included holistic assessment, opportunity for end of life discussion, symptom management and carer reassurance. Older people and carers advocated early access to SIPS, while other stakeholders proposed delivery based on complex symptom burden. A priority for integrated working was the assignment of a key worker to co-ordinate care, but the assignment criteria remain uncertain.

**Interpretation:**

key stakeholders agree that a model of SIPS for frail older people with non-malignant conditions has potential benefits within community settings, but differ in opinion on the optimal timing and indications for this service. Our findings highlight the importance of consulting all key stakeholders in model development prior to feasibility evaluation.

## Introduction

The ageing population and associated rise in long-term conditions present challenges to established models of care for older people [1, 2]. People are living longer and increasingly with multi-morbidity and frailty. Frailty is defined as the accumulation of deficits and diminishing reserves [3]. This increases vulnerability to a seemingly minor stressor event leading to a marked deterioration in well-being and poor outcomes [3, 4]. The frailty state is characterised by an illness trajectory of prolonged dwindling with intermittent episodes of decline [5]. Frail older people commonly experience high physical and psychological symptom burden, which is frequently under-reported and under-treated [6].

Palliative care aims to relieve suffering and improve quality-of-life for patients and their families through holistic assessment of physical and psychosocial problems associated with life-threatening illness [6]. Palliative care is delivered in all care settings by both specialists, who focus on patients with advanced illness and complex problems, and generalists where palliative care is part of their clinical role, e.g. general practitioners [7]. Specialist palliative care has historically been offered to patients with cancer; however, its value to those with non-malignant conditions is increasingly recognised. These groups have comparable levels of need to people with cancer [8] and are at risk of poor outcomes, e.g. distressing symptoms or social isolation [6].

Understanding how best to provide palliative care to frail older people is an international priority [9]. There is no clearly transferrable model of specialist palliative care as the majority of evidence concerns cancer [10] or non-malignant conditions in isolation [11]. However, components of these models might inform aspects of a service for frail older people with non-malignant conditions. For example, *episodic* involvement of specialist palliative care is advocated for patients with non-malignant conditions. Specialist palliative care is proposed in response to exacerbations of disease in chronic respiratory conditions [12], and Higginson *et al* demonstrated the benefit for patients with multiple sclerosis and their carers [13]. Such periodic engagement may be applicable for frail older people; however, exacerbations or episodes of decline are heterogeneous and unpredictable, leading to uncertainty about the indicators for referral to specialist palliative care [5]. Moreover, for continuity of care it is essential to work with existing providers of care to older people, notably general practitioners and social care staff as the main providers [6].

We sought to build on understanding of episodic palliative care and increase the specificity of this model for frail older people in community settings. We proposed a model of short-term integrated palliative and supportive care (SIPS) for frail older people aged 75 years or over with non-malignant conditions living at home or in a care home. The initial model comprised one to three contacts with the specialist palliative care team and integrated professional working between specialist and existing generalist providers. We aimed to build consensus on three key components of the model: potential benefit; timing of delivery and integrated professional working practices. Objectives were to elicit and synthesise perspectives from older people, carers and other key stakeholder to inform model development prior to a feasibility evaluation in clinical practice [14].

## Methods

Observational study design that drew on the Medical Research Council's guidance on developing and evaluating complex interventions [14]. Following this guidance, we systematically developed the SIPS intervention. Higginson *et al*’s work on short-term palliative care informed the initial model [13]. We engaged service users and providers to adapt the model for frail older people by examining the intervention processes and the intended outcomes [14]. The study was approved by the London- Queen Square NHS Research Ethics Committee (REC reference 13/LO/1304).

**Figure 1.**
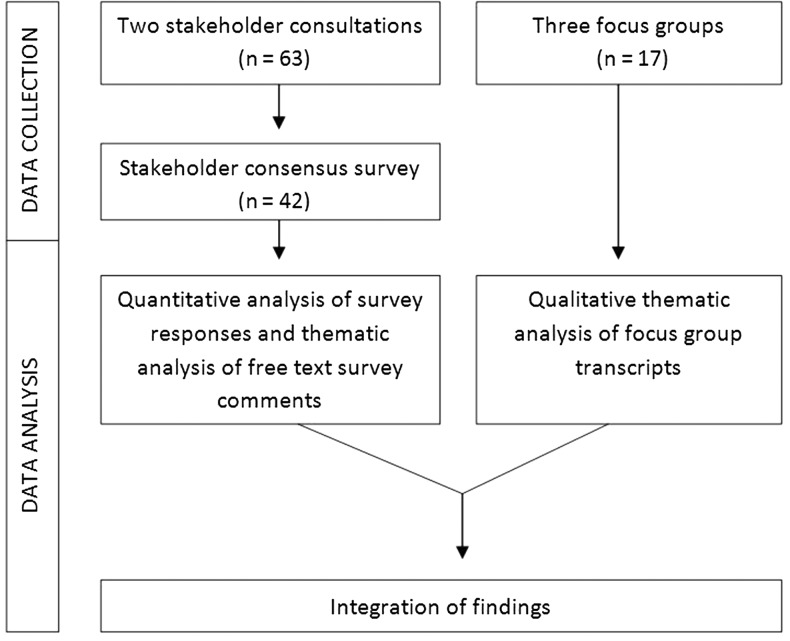
Schematic of data collection and analysis.

The observational design incorporated transparent expert consultations using a modified nominal group technique to generate recommendations, and a follow-up online consensus survey to examine recommendation priorities [15]; and focus groups with older people and carers to deepen the understanding. The structured nominal group technique allowed rapid generation of recommendations from a range of stakeholders through group discussion and prioritisation, while minimising researcher bias [15, 16]. Focus groups were an effective and enabling way of exploring sensitive issues and eliciting views of patients and carers [17]. The consultations and focus groups were conducted in parallel (January and February 2014), and the online consensus survey followed (closed May 2014) (Figure 1).

### Setting

The study was conducted in two contrasting geographical areas in Southern England (rural/urban and city) with a total population of over one million and geographical area of 800 square miles [18]. The rural/urban area has a higher proportion of deaths occurring at older ages (age 75 or over) than that of England and Wales, 74% and 68%, respectively, while the proportion in the city area is comparable to national figures [19].

### Stakeholder consultations and consensus survey

Two consultations, each lasting 4 h, were held in a community facility and an NHS building in each area. Participants were purposively sampled to maximise the range of perspectives from service providers (generalists and specialists from health and social care), unpaid carers, service commissioners for end-of-life care, voluntary sector representatives, researchers and lay members supporting the research study. The service providers and commissioners were identified by clinicians in the study site and members of the research group. Our independent project advisory group, comprising lay members, assisted with recruitment by identifying local and national charitable organisations that support older people. An invitation letter and participant information sheet was sent or emailed to individuals or organisation leads for distribution. All participants gave informed written consent prior to commencement of the consultation.

The consultations opened with presentations and discussion on the findings from the preceding intervention development work (which involved a mortality follow-back survey on preferences and outcomes of care for older people at the end-of-life [20]), and the proposed SIPS intervention. Participants worked in three groups comprising a range of disciplines, with a facilitator and scribe. All facilitators were co-applicants on the study and health professionals, including both specialist palliative care and generalist professions, with expert knowledge of the field (C.E., S.T., F.L. and C.B.). The range of health professions was designed to minimise the extent to which facilitators would influence discussions in a particular direction. Each group focused on one of the three main areas:

1. What are the potential benefits of short-term integrated palliative care?
2. When is the optimal time to provide this service?
3. How should integrated care between specialist palliative care and generalist community and primary care services be delivered?

Facilitators guided participants through a modified nominal group process of brief discussion, individual writing of recommendations and rationale on structured sheets, and reading out of recommendations until individual lists were exhausted. Each group discussed the recommendations, and agreed the final priority order. All participants reconvened to discuss the top three recommendations from each group. Discussions were digitally recorded and key points transcribed. Recommendations were combined and duplicates removed by two researchers (A.B., C.E.) to generate a final set of recommendations for each key area. All participants received the final recommendations in an online survey (KeyPoint version 6, Speedwell Software, Cambridge, UK) or printed with a pre-paid return envelope when required. Participants were asked to rate the level of importance of each recommendation (1: low to 9: high) and to provide free text comments on the rationale for their decision.

### Focus groups

The focus groups intended to enrich the consultation findings by eliciting perspectives of older people and carers. We purposively selected existing groups comprising people living with frailty or supporting those living with frailty. The study's independent project advisory group and steering group identified potential groups. The selection of existing groups intended to provide a familiar and supportive environment for the participants and enable their involvement [17]. Three focus groups and their settings comprised:

- Older people residing in a nursing home, conducted in the nursing home.
- Day centre attendees and informal carers, conducted in the day centre.
- Volunteer carers from a charitable organisation supporting older people living alone in the community, conducted in an NHS building.

The managers of the respective organisations gave eligible participants an information sheet on the study and an invitation for the focus group. One researcher (C.E.) visited older people in the nursing home and day centre to explain what participation involved. Informed consent was undertaken by skilled research nurses and researchers (C.E., A.B.), with participants in their room in the nursing home, or in a quiet space in the day centre. Participants gave written consent or verbal consent with a witness signature if visually impaired. The volunteer carers received an email and/or a telephone call from a researcher (A.B.) to discuss the study, and completed the informed consent process on arrival for the focus group.

The focus groups, each lasting 90–120 min, were digitally recorded. Discussions were facilitated by an experienced clinician and qualitative researcher (C.E.) with an observer (A.B.). Given the sensitive nature of the topic, the study incorporated a distress protocol. The facilitator (C.E.) began the focus group by detailing the intention of the discussion and clarifying that an individual could stop at any point, and a member of staff was available to provide support e.g. care home staff. To stimulate discussion in a sensitive way we used vignettes that intended to resonate with older people and carers’ lived experiences. The vignettes focused on the three key areas of the SIPS model using a scenario of an elderly man with increasing frailty and hospital attendance, and his family (box S1 in Supplementary data are available in *Age and Ageing* online). Vignettes are considered helpful to facilitate discussion on sensitive issues e.g. dementia care [21]. The vignettes were informed by findings from the earlier post-bereavement survey [20], and the language and content refined with support from the independent project advisory group and steering group. After the focus group, informal time was provided over refreshments for participants to ‘de-brief’ on taking part, and a follow-up contact with the researcher (C.E.) was offered to discuss issues arising from participation.

### Data analysis and integration

Quantitative consensus survey data was summarised using descriptive statistics e.g. median and interquartile range. Importance ratings from one to nine for recommendations were assigned to pre-defined categories of indication: *indicated* (median 7–9), *equivocal* (4–6) or *not indicated* (1–3) and consensus: *strict agreement* (interquartile range was within a three-point region) or *broad agreement* (interquartile range exceeded a three-point region) [22]. Free text responses were collated in Excel to explore the issues raised for each recommendation.

Focus group discussions were transcribed verbatim and anonymised using identification codes for all identifiable data. Transcripts were analysed in NVivo 10 (QSR International, Victoria Australia) using a directed content analysis approach [23]. The top-level structure of the coding scheme was determined by the three key areas of the SIPS model explored across the groups, and themes within each area were derived from the data. Additional codes were formed for emergent themes [23]. Transcript coding was conducted by two researchers (A.B., C.E.) with disagreements about assignment resolved through discussion. The consensus survey and focus group findings were integrated at the point of analysis using triangulation [24]. All data were categorised according to the key areas. We identified convergence and divergence within and between the two data sets.

## Results

### Participants

There were 80 participants including older people and informal carers, generalist providers (e.g. general practitioners (GPs) and district nurses) and specialist providers (e.g. consultants in palliative medicine and nurse specialists in palliative care), health service commissioners, representatives from the voluntary sector and social care and researchers (Table 1). Sixty-three people participated in the stakeholder workshops and 60% completed the consensus survey with additional responses from stakeholders who registered interest in the workshops but were unable to attend. Seventeen people (13 women) participated in the focus groups, including volunteer carer participants (*n* = 7), day centre attendees (*n* = 2), attendees’ informal carers (*n* = 2) and older nursing home residents (*n* = 6) (Table 1).

**Table 1.** Stakeholder consultations, consensus survey and focus group participants’ roles

Participants’ role	Stakeholder workshops and consensus survey		Focus group discussions
	Consultations	Consensus survey	
Nursing home resident	–	–	6
Day centre attendee	–	–	2
Informal carer	4	2	2
Volunteer carer	2	0	7
General practitioner	5	5	–
Community nursing service	11	7	–
Palliative medicine consultant	4	3	–
Specialist palliative care nurse	7	4	–
Other specialist nurse e.g. heart failure	3	3	–
Hospice e.g. education and management leads	2	1	–
Allied health professional	0	1	–
Care home e.g. manager	1	1	–
End-of-life care commissioner	1	0	–
Voluntary sector representative e.g. Alzheimer's Society	12	8	–
Academic/researcher	11	7	–
			
**Total**	63	42	17

### Stakeholders’ recommendations and focus group themes

The stakeholder consultation generated 473 items to be considered for recommendations within the three key areas. Thirty recommendations were included in the consensus survey. The importance of each recommendation was *indicated* (median rating: 7–9), with *strict agreement,* except for two recommendations with *broad agreement* (Figures S2–4 in Supplementary data are available in *Age and Ageing* online). Nine recommendations concerned potential benefits of the SIPS model for patients, e.g. information for decision-making or symptom management, and/or carers, e.g. bereavement support. Eight recommendations concerned timing of SIPS delivery, e.g. symptom burden, and 13 recommendations concerned integrated working, e.g. single contact for specialist palliative care advice.

**Figure 2.**
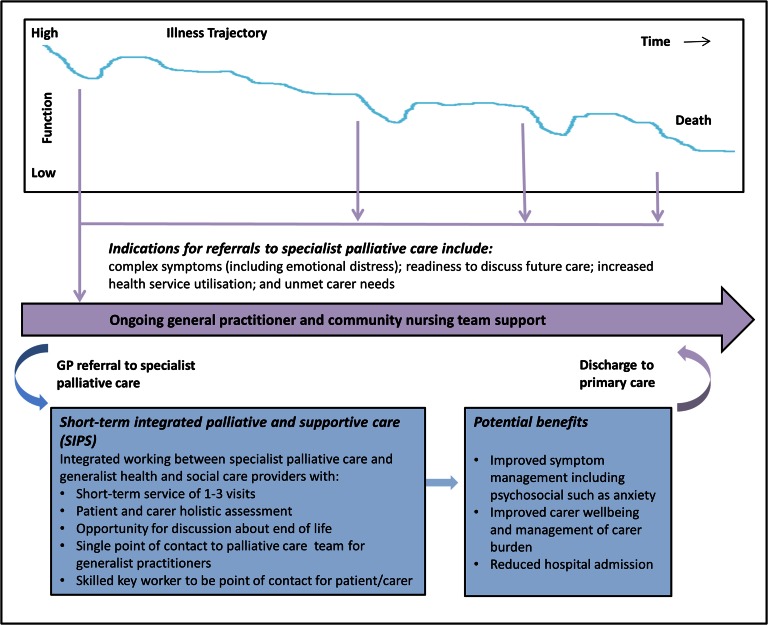
Model of Short-term Integrated Palliative and Supportive Care (SIPS).

The main themes from focus group discussions were potential benefits ‘being heard’ and ‘reassurance for carers’; timing of delivery ‘points of downward spiral’ and integrated working practices ‘a community link person’. Stakeholder recommendations were integrated with the themes from the focus groups (Table 2) and were used to develop a SIPS model for frail older people with non-malignant multi-morbidities (Figure 2).

**Table 2.** Stakeholder recommendations and themes across the focus group discussions and consensus survey free text comments

**What are the potential benefits of short-term integrated palliative care for the frail elderly with non-malignant conditions?**	
**Recommendation 1:** **To ensure older people are better informed about choices with opportunities to plan future care and to be empowered to make decisions (median: 9, IQR: 8–9)**	
**Holistic discussions**	*Everything, across the board. They've got a particular thing like back pain, they need someone that really understands back pain. If they've got a spiritual problem, they really need someone that is like neutral and can share whatever. (FG-VC6)*
	*Extremely important because it allows individuals to take control of their lives and that is crucial for mental wellbeing. (CS-VSR)*
**End-of-life discussions with skilled professionals**	*But maybe have that additional expertise about being able to raise the issues of end-of-life, based upon their experience and things. (FG-VC1)*
	*Offer them* [the older person] *the choice. Because there's a certain pride and independence still and dignity that they are not being brushed to one side and being dealt with in the same way as everyone else; that they're being heard”. (FG-VC5)*
	*The more information offered to the family and older patient/person can only be to everyone's advantage. To ensure this is given at the right juncture and carried out sensitively would prevent awkward hurried conversations or worse still making decisions on behalf of the person's future care without previous knowledge of their wishes such as patients with memory loss or cognitive impairment. (CS-CN)*
	*Could I just say, just with dying, I mean surely there must be different strategies for confronting your own mortality? Some people won't ever want to be confronted with it…Other people actually will want people much more practical about it and recognise the things, wanting the people to acknowledge them…But within this set of people dealing with this situation, there needs to be somewhere the capacity to confront those different strategies to know when it's appropriate to talk about the things. (FG-VC4)*
**Recommendation 3:** **To increase carers’ sense of reassurance and support by assessing a carer's needs, identifying the assistance required and involving them in discussions about plan of care (median: 8, IQR: 8–9)**	
**Inclusion of carers in discussions and reassurance**	*…if it comes to a situation also where someone may be dying, if you're [carer] somehow not included in it, then you could feel not being able to grieve properly. (FG-VC1)*
	*Hopefully the integrated service will be inclusive of carers - who may not share views on ‘preferred place of care’ and who may need reassurance about the levels of care and help that might be required in caring for someone at home. (CS-SN)*
	*His wife needs reassurance and support and the daughter needs to be kept in the loop to know what's going on because she feels, obviously, her contribution is limited because she's got her family to take care of. But still, she will still be worrying though she's not hands-on. She would still be anxious and worried, so she needs support. She needs the reassurance that her parents are both getting the help that they need. (FG-IC1)*
**Carer well-being and enabling carers to care**	*Carers who fall ill because of stress will not be able to fulfil their role, and it is therefore important to support them. (CS-IC)*
	*Sometimes I think, ‘Oh, you must go down there. You must do this,’ and I think, ‘No, I've got to go out for the day and I've got to look after my mind because I shall lose my mind otherwise with it,’ because you could drown in it. You absolutely drown in it. (FG- IC2)*
	*I kept going to his oncologist. I didn't go in with him but I wrote to him in the end and I said…well really, it was a letter of plea saying, “I just don't know what to do.” (FG-NH5)*
**Carer as advocate for older person**	*….Nothing will take the place of a loyal family member. That is gold dust, and you will never replace that, but unfortunately not everybody's got a family member. (FG-IC2)*
	…*if any of us get to the situation of Mr. Wood* [in the vignette]*, what we need if we're lucky, is someone who's looking out for us. In his case it could be his daughter. Someone who will go to the hospital or go to the surgery and knock on the door until somebody does something”. (FG- VC2)*
**When is the best time to refer frail older people to specialist palliative care?**	
**Recommendation 16:** **When an older person is living with increasing symptom burden (including emotional/mental), which the community nurses/GP are struggling to manage, or are causing concerns about planning future care (median: 9, IQR: 8–9)**	
**Accumulation of multiple problems**	*The service would be extremely beneficial to those with complex needs and symptoms where generalist health care professionals may be having difficulty in managing these needs. (CS-A)*
	*…people that really need palliative type care usually have multiple conditions. It could be heart and depression. It could be like deafness, blindness but they all have multiple things going on. (FG-VC6)*
**Mental frailty and emotional support required**	*Physically/medically ill patients are within my remit but it would be a helpful tool for me and my team to be able to refer on if we had a patient who required emotional or mental support. (CS-CN)*
	*There's also a mental frailty, a loss of confidence. So when you may have had a small health problem, getting back over it again, not only physically to recover but a loss of confidence in doing things that you want to, and going out may be or even within your flat.(FG-VC7)*
**Recommendation 10:** **Early when an older person is increasingly struggling to manage at home because they live alone/housebound (median: 8 IQR: 7–8)**	
**Improves familiarity and continuity with the team**	*“[Receiving specialist palliative care later] I think they'd be more resistant, whereas I think if they'd already had that first input they would familiar; Oh yeah, we need a bit of extra help. We had that last time.” (FG-VC6)*
**Discussing end-of-life earlier to anticipate and prepare**	… *it just occurs to me that some of the things, conversations about dying or what you'd want, much better conducted when you're not really going about to die. (FG-VC1)*
	*Listening at the right time when you're able to talk and you haven't got the pressure when you're just feeling okay but you can talk. Who is going to talk sense when we've got crippling back pain? You're just going to want solutions but people just are not being heard and that's part of the lack of respect that we're getting in society. No one has the time to actually hear you and that's what people need. (FG-VC6)*
**SPC may not be required earlier**	*May not require specialist input at this point. Needs should be assessed by primary care team. (CS-PMC)*
**How to best deliver integrated care between specialist palliative care and the primary health care team?**	
**Recommendation 18:** **Fast and easy access to specialist palliative care advice and support through a single phone contact point (median: 8.5, IQR: 8–9)**	
**Importance of fast effective treatment**	*For the person/carer as well as other health care professionals. (CS-VSR)*
	*Fast effective treatment is of the utmost importance. (CS-CN)*
**Out of hours access**	*I mean, you can't just ring an ambulance and say…the paramedics are marvellous but can't be ringing them twice a day and saying he can't breathe because they can't do anything more. (FG-NH6)*
	*This access needs to be well publicised and available 24 hours a day, as crises usually occur at night, or out of office hours. (CS-IC)*
	*Well hopefully, some degree of accessibility when he needs weekend attention…when he can't access his GP. (FG-VC3)*
**Recommendation 20:** **Co-ordination of services through a key worker from the SPC team acting as a single point of contact for the patient and family to avoid confusion and maintain continuity across care services (median: 8, IQR: 7–9)**	
**Key worker assigned to patient for continuity of care**	*Yes. Too often patients just don't know to whom to turn. (CS-VSR)*
	*The service still requires very good communication between ALL teams, as one person missing, through sickness or leave, should not mean that the patient and family have no-one to turn to. (CS-IC)*
	… *there needs to be someone there saying, “This is my person”. (FG-VC1)*
	*If there is always that person in their life, so if there is a support and it's always around then when that support walks up to see them when they're facing a crisis or got to make a decision, in their mind they're like, ‘Oh, it's all right. [Clinician's name]’s here. All right, now,’ in their mind because they know that they can trust what you say. (FG-IC1)*
	*Because I think older people are very much aware of continuity. Old age doesn't like too much change and that's where a lot of care falls down. The continuity is simply not there. (FG-VC3)*
**Uncertainty over who should be responsible for key worker role**	*V important but should be the most involved member of the team/the one with the relationship which may or may not be specialist palliative care. (CS-H)*
	*No. The key worker must come from primary care. (CS-PMC)*
	*Co-ordination could be via GP still if only limited visits anticipated. Who to contact and when should be part of future care planning and information given to carers. (CS-SPC)*
	*This* [a key worker] *is extremely important but does the key worker need to be from the specialist palliative care team? Shouldn't it be the service/person who knows the patient and has the most contact? (CS-SPC)*
**Concerns over short-term nature of SPC providing this role**	*Yes, but I'm not sure that this will be effective for short-term input only—the single point of coordinated contact needs to be long standing to be effective. (SC-GP)*
	*But could be problematic especially as this is a short-term service. (SC-A)*
	*I think this could add to confusion. (CS-CN)*

Participants’ ID codes are prefixed by the data collection method (CS for consensus survey free text comments or FG for focus group discussions) and suffixed by their role (VSR,  voluntary sector representative; VC,  volunteer carer; CN,  community nurse; IC,  informal carer; NH,  nursing home resident; SN,  other specialist nurse, e.g. heart failure; A,  academic/researcher; PMC,  palliative medicine consultant; GP,  general practitioner; H,  hospice e.g. education and management leads; SPC,  specialist palliative care nurse). IQR,  interquartile range.

#### Potential benefits: ‘being heard’ and ‘reassurance and support for carers’

A main benefit of the involvement of palliative care centred on ensuring older people were better informed about choices with opportunities to plan future care (recommendation 1, median: 9, IQR: 8–9). Survey respondents commented that this was important to empower patients, promote mental well-being and to ensure patient-centred care.

‘Not knowing what is happening in terms of care in the present and near future is extremely distressing both for patient and family—undue additional stress at an already stressful time’ (Consensus survey—VSR).

The focus group theme of ‘being heard’ and not ‘brushed to one side’ identified the imperative of a holistic assessment that encompassed the range of health and social challenges encountered with advanced frailty for the older person and their carer. A vital component was providing opportunities to discuss and plan future and end-of-life care, which in turn foster involvement in decision-making and promotes individual's dignity. However, it was noted that enabling discussion on sensitive issues requires professional skill and expertise to *“give* [a person] *the* *opportunity to start the conversation about the last part of their lives”* (Focus group—VC1).

Stakeholders placed high importance on the benefit of assessing carers’ needs to identify the assistance required and provide reassurance and support (recommendation 3, median: 8, IQR: 8–9). Inclusion of carers in discussions formed a prominent theme in the focus groups. Having someone for a carer to talk to about their worries was important to acknowledge the ‘stress and strain’ of caring and their frequent social isolation, and provide emotional reassurance. Supporting carers was an imperative to maintain their well-being and enable them to continue their caring role, particularly with increasing care needs for the older person towards the end-of-life.

‘Respite for carers if needed is of prime importance. Supporting carers, as without them the patient would not be able to stay in their home if their wishes are to stay and end their life [die] at home’ (Consensus survey—CN).

A prominent theme across the focus groups, but little discussed in the stakeholder consultations, was the importance of a family member who could advocate for an older person ‘somebody to speak for you’. Participants identified the increasing challenge of older people living in social isolation and their vulnerability to poor health with no one to advocate on their behalf. This placed increasing emphasis on voluntary services to maintain and promote quality-of-life and co-ordinate formal services.

‘In a way for my job [as a volunteer carer], I find it quite surprising that…you know, we're just volunteers, that so many with the [Volunteer Carer] Scheme, with the volunteers, you could actually [be]…the main people looking out for really vulnerable people who aren't getting the support that they need’ (Focus group—VC1).

#### Timing of delivery: ‘progressive frailty and downward spiral’

Stakeholders agreed that a key indication for referral to palliative care was when an older person had increasingly complex symptom burden (recommendation 16, median: 9, IQR: 8–9). These were points when generalist care-providers “struggled” to relieve symptom distress (including physical, emotional or mental) or plan future care.

‘I'm not sure about resource implications but I would think that a specialist palliative care service would need to focus more on complex symptom management’ (Consensus survey—A).

Participants highlighted the complexity of care management for frail older people when living with multi-morbidity, and the inter-relatedness of an older person's physical, psychological and social presentation and the progressive and unpredictable nature of frailty.

‘Well I was going to say all these frailties is progressive. You've only got to trip up and feel tired and then you don't make your meal that evening and then you become physically less able to do things. And it's just progressive. Just one trip will, or one anything, will set the whole of that off, and it is the downward spiral’ (Focus group—VC4).

However, the addition of palliative care was also recommended ‘early’ in an older person's illness, particularly when they lived alone and increasingly struggling to manage at home (recommendation 10, median: 8, IQR: 7–8). This disparity reflected the different perspectives of the participants. Practitioners advocated requirement for assessment and support from generalist providers and addition of specialist palliative care if there was complex symptom presentation. In contrast, a key theme for older people and carers in the focus groups was referral to palliative care early in the illness trajectory. Participants suggested that early referral would improve familiarity and continuity with the palliative care team and allow the older person an opportunity to make informed end-of-life decisions while they had cognitive capacity and before their condition deteriorated.

‘They would be more prepared. They would have to think about it, whereas they put it to the back of their mind until it happens’ (Focus group—NH2).

#### Integrated working: ‘community link person’

A key component of integrated professional working between specialist and generalists was fast and easy access to specialist palliative care for advice and support (recommendation 18, median: 8.5, IQR: 8–9). Stakeholders considered a single phone contact point for specialist palliative care as essential to facilitate communication between the generalist community services and the specialist palliative care team, particularly out of hours. Stakeholders recommended identification of a skilled key worker from the palliative care team to act as a single point of contact for patients and carers, and to co-ordinate services and care (recommendation 20, median: 8, IQR: 7–9). Older people and carers in the focus groups identified the importance of a named lead for a person's care to foster continuity and build trust between the older person and the individual to support decision-making on care and treatment.

‘….they need one community link person saying, “So and so is in charge and this person will be doing this,” but it all comes to one person that they can make a sort of link with and they can trust, and they know whatever their range of problems is, it's being sorted by someone who is overviewing the whole case’ (Focus Group—VC6).

However, uncertainty surrounded, which professional should be assigned the key worker role, for example, palliative, primary or social care. It was suggested that the key worker could be from the service with the greatest involvement with the older person or could vary depending on patient need and corresponding service involvement. The proposed short-term episodic nature of the SIPS model was considered likely to limit the effectiveness of members of the palliative care team acting as the key worker to co-ordinate and review ongoing care.

## Discussion

We integrated the views of older people, carers and other key stakeholders to develop a model of short-term integrated palliative and supportive care (SIPS) for frail older people with non-malignant conditions living in the community (Figure 2). Consensus was established regarding key components of the model. SIPS should aim to improve symptom management, encompassing physical and psychosocial distress, facilitate end-of-life discussions, and reduce carer burden and unplanned hospital admissions. Patient referral was advocated at multiple points, both early in a patient's illness trajectory when vulnerable to marked decline or loss of mental capacity and also during episodes of decline and complex symptom presentation. Priorities for integrated working comprise a single point of access for palliative care services, and a skilled key worker identified from the service with greatest patient involvement to co-ordinate care.

Older people and carers viewed specialist palliative care as an acceptable ‘additional layer of support’ that could facilitate discussions on advanced care planning and improve outcomes of care for patients and carers through comprehensive assessment and specialist skills in complex symptom management. Studies have found that frail older people have little understanding of likely deterioration of their condition [25] and discussing care preferences in advance can enhance end-of-life care, e.g. dying in the preferred setting [20]. The invaluable contribution of carers as advocates highlighted the relevance of timely care planning for older people who live alone and do not have ‘somebody to speak for them’ should they deteriorate and lose capacity. The identification of frail older people who could benefit from palliative care is challenging as the indicators for referral are poorly established [26]. Evidence supporting early palliative care can lead to improved quality-of-life and potential cost savings [27]. Older people and carers advocated earlier referral to allow for timely discussion of end-of-life issues and for relational continuity. More evidence on cost-effectiveness of palliative care for frail older people is required to inform the allocation of resource towards the speciality, to enable early access where warranted [28].

The importance of continuity and co-ordination of care to patients and carers is well-established [29]; however, the episodic and short-term nature of the SIPS model presents challenges to these ideals. An integrated approach could enable continuity of care from the main generalist provider, develop generalists’ provision of palliative care through education and support and direct specialist care at points of increased need. The benefit of having a clear point of contact with a familiar healthcare professional, who oversees and co-ordinates different services was emphasised. A nominated key worker was advocated; however, there was concern about this being a member of the specialist palliative care team due to their short-term involvement. In line with other reports [30], out of hours access to specialist care through a single contact number was considered important to meet patient and carers’ needs for information, and to provide reassurance during crises. How this might prevent unplanned hospital attendances requires further study.

This study incorporated a wide range of perspectives, including those from older people and their carers, which are frequently omitted from model development [14]. Eliciting views from all key stakeholders, establishing consensus using a follow-up survey, and triangulating findings with in-depth focus group discussions, increased our confidence in the final SIPS model. The divergence of opinion on some issues highlighted the importance of including all key stakeholders in service development. There are also some limitations to consider. Selection bias was possible as those with an interest in older people and end-of-life care were more likely to participate, excluding those less engaged from the discussion whose views may differ. Nonetheless, there was disagreement among participants, for example, about which team the key worker should be based, demonstrating some balance of overall opinion. The consensus survey was conducted in a single round. A multi-round Delphi process could have further refined the recommendations. However, we did find a high level of agreement between participants regarding the importance of each recommendation.

Improved understanding of how to provide palliative care to the growing number of older people living with frailty, multi-morbidity and nearing the end-of-life is an international priority [6]. The SIPS model has been carefully developed in consultation with key stakeholders. There is strong consensus among stakeholders that an episodic SIPS model has significant potential to benefit older people and their carers in community settings. Differing views on when specialist palliative care should be delivered demonstrate a need to balance offering older people and carers SIPS early in the illness trajectory, with the reality of finite resources and growing demand. There was an agreement that a nominated key worker is required to co-ordinate care, but questions remain about how this would work in practice. The model now requires formal feasibility testing before its effectiveness can be determined.

**Key points**

- Understanding how best to provide palliative care to frail older people with non-cancer conditions is an international priority.
- We proposed an episodic model of short-term integrated palliative and supportive care (SIPS) for this group.
- We elicited a wide range of perspectives, including those from older people and carers, on key components of the model.
- Potential benefits of SIPS include complex symptom management, advanced care planning and carer reassurance.
- Timing of SIPS delivery must balance the possible benefits of early intervention with the reality of finite specialist resource.

## Supplementary Material

Supplementary Data
