# Supplementary material for: Developing a model of short-term integrated palliative and supportive care for frail older people in community settings: perspectives of older people, carers and other key stakeholders
Source: Age Ageing. 2016 Nov 2;45(6):863–73. doi: 10.1093/ageing/afw124 (PMC5105822; doi:10.1093/ageing/afw124)
Supplement: Supplementary Data [file supp_45_6_863__index.html]

Developing a model of short-term integrated palliative and supportive care for frail older people in community settings: perspectives of older people, carers and other key stakeholders — Developing a model of short-term integrated palliative and supportive care for frail older people in community settings: perspectives of older people, carers and other key stakeholders — Developing a model of short-term integrated palliative and supportive care for frail older people in community settings: perspectives of older people, carers and other key stakeholders — Supplementary Data 

# Developing a model of short-term integrated palliative and supportive care for frail older people in community settings: perspectives of older people, carers and other key stakeholders

## Supplementary Data

Supplementary Data

- Supplementary Data - docx file
