# Supplementary material for: Developing a model of short-term integrated palliative and supportive care for frail older people in community settings: perspectives of older people, carers and other key stakeholders
Source: Age Ageing. 2016 Nov 2;45(6):863–73. doi: 10.1093/ageing/afw124 (PMC5105822; doi:10.1093/ageing/afw124)
Supplement: Supplementary Data [file supp_afw124_aa-16-0032-File001.docx]

**Supplementary Data**

**Suppl. Box S1:**  **Focus group Vignettes and Topic Guide**

**Vignette 1**

Mr Wood is an 88 year old man with mild memory problems. He comes to the Accident and Emergency Department with his wife. He has terrible back pain because of arthritis in his back. Mr Wood has been taking a lot of paracetamol as it only eases his pain a little bit.

Mr Wood has come into hospital four times over the last six months because of pain, falling, becoming more confused and losing weight.

His elderly wife, Mrs Wood is finding it very hard to support and care for her husband. Their daughter Sarah lives nearby and is trying to help her parents, but works part-time and has a young family.

**Mr Wood says:**

*I told the doctor that I never want to go to the hospital again. It’s torture—you can’t do anything for yourself and you get weaker and sicker. Every time I’m in the hospital it feels as if I’ll never get out.*

**Mrs Wood says:**

*He hates being in the hospital, but what can I do? The pain is terrible and I can’t get the Out of Hours doctor to call me back. I can’t even move him myself, so I call an ambulance. It’s the only thing I can do.*

**Sarah says:**

*I want what dad wants, which is for him to stay at home. But Mum can’t manage when Dad is in pain. She doesn’t know what to do. It is very distressing for her when she is struggling to get someone to help Dad.*

**Vignette 2**

Three months have passed. Mr Wood is much frailer. He needs help from his wife to get up from bed, to walk to the bathroom, to wash and dress and manage his medicines. He is spending more and more time in bed resting and sleeping. He still enjoys a little food and having his wife close. He is more confused and relies on his wife to help explain things and remind him what he needs to do.

He comes to the Accident and Emergency Department with his wife because he has a chest infection. His GP prescribed an antibiotic, but his chest has not got better. This is the second time in two months that Mr Woods has come to hospital with a chest infection.

**Mr Wood says:**

*I told the GP I did not want to go back into hospital again. It’s so tiring and noisy. I don’t know who people are and I miss my wife, but it is difficult for her to get here. Each time I go into hospital I want to get home again, but it gets harder each time to get home. I want to stay at home next time.*

**Mrs Wood says:**

*I don’t know what to do. His breathing is so bad, it is so frightening. I can’t get hold of the doctor on the weekend. I don’t want him back in the hospital, he hates it, but what can I do? I call the ambulance. It’s the only thing I can do.*

**Sarah says:**

*Mum panics, she always calls the ambulance. Dad saw the doctor and he explained he could stay at home. The doctor explained that Dad might not get better with the antibiotics at home, and that he could arrange for nurses to help look after dad at home. He also said that Dad could take medicine to help his breathing. That’s what he wants, he wants to stay at home with more support, but Mum panics.*

**Questions:**

1. What do you think is important to Mr Wood and his family at this time?
2. What do you think about the GP suggesting that the palliative care get involved at this time?
3. How might this look different if there was no Mrs Wood or Sarah and Mr Wood lived alone?

**Suppl. Figure S2: Stakeholder recommendations for potential benefit of SIPS and consensus survey findings (mean, range and interquartile range)**

**
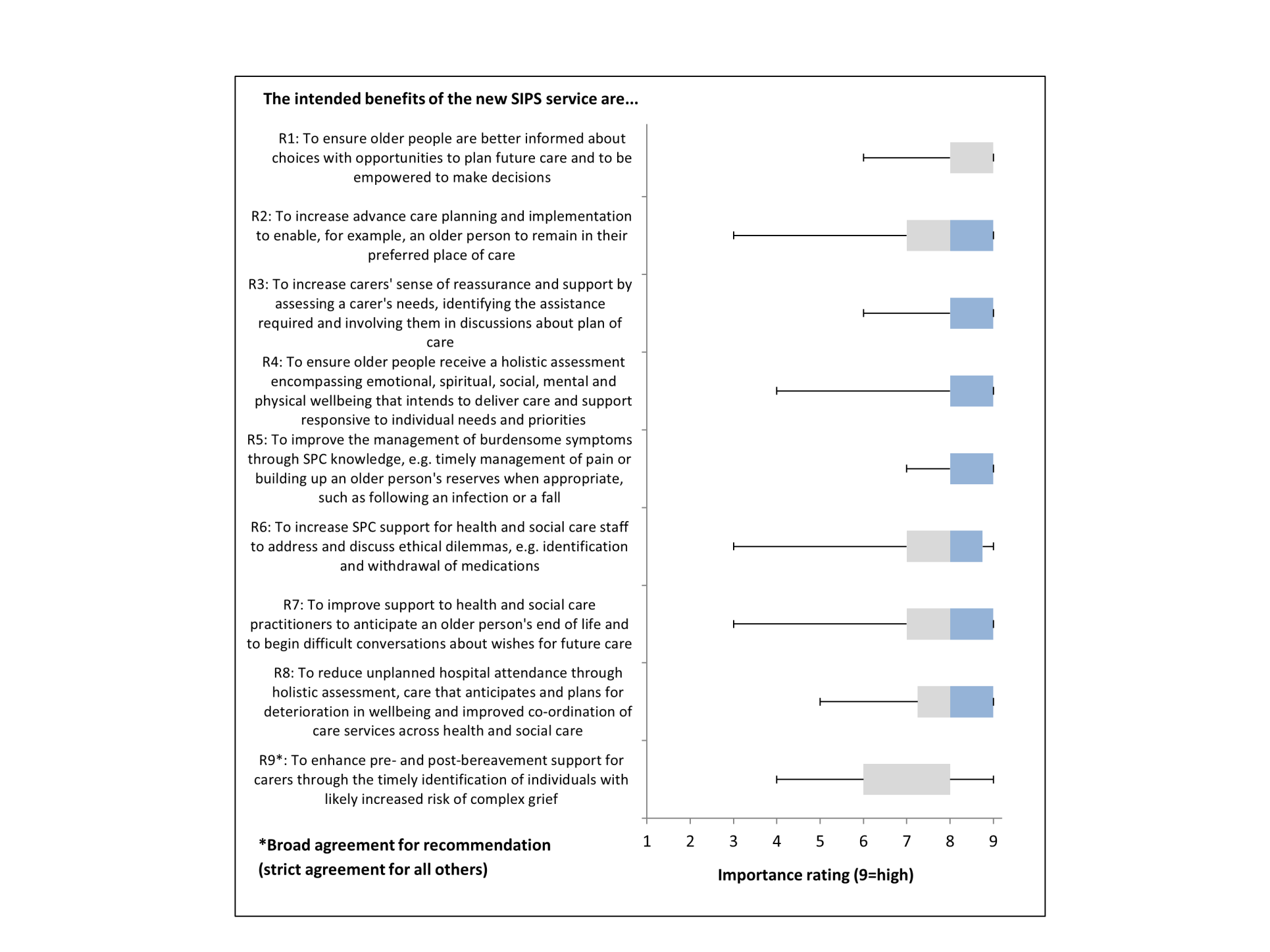
**

**Importance rating (9=high)**

***Broad agreement for recommendation (strict agreement for all others)**

**Suppl. Figure S3: Stakeholder recommendations for timing of SIPS delivery and consensus survey findings (mean, range and interquartile range)**

**
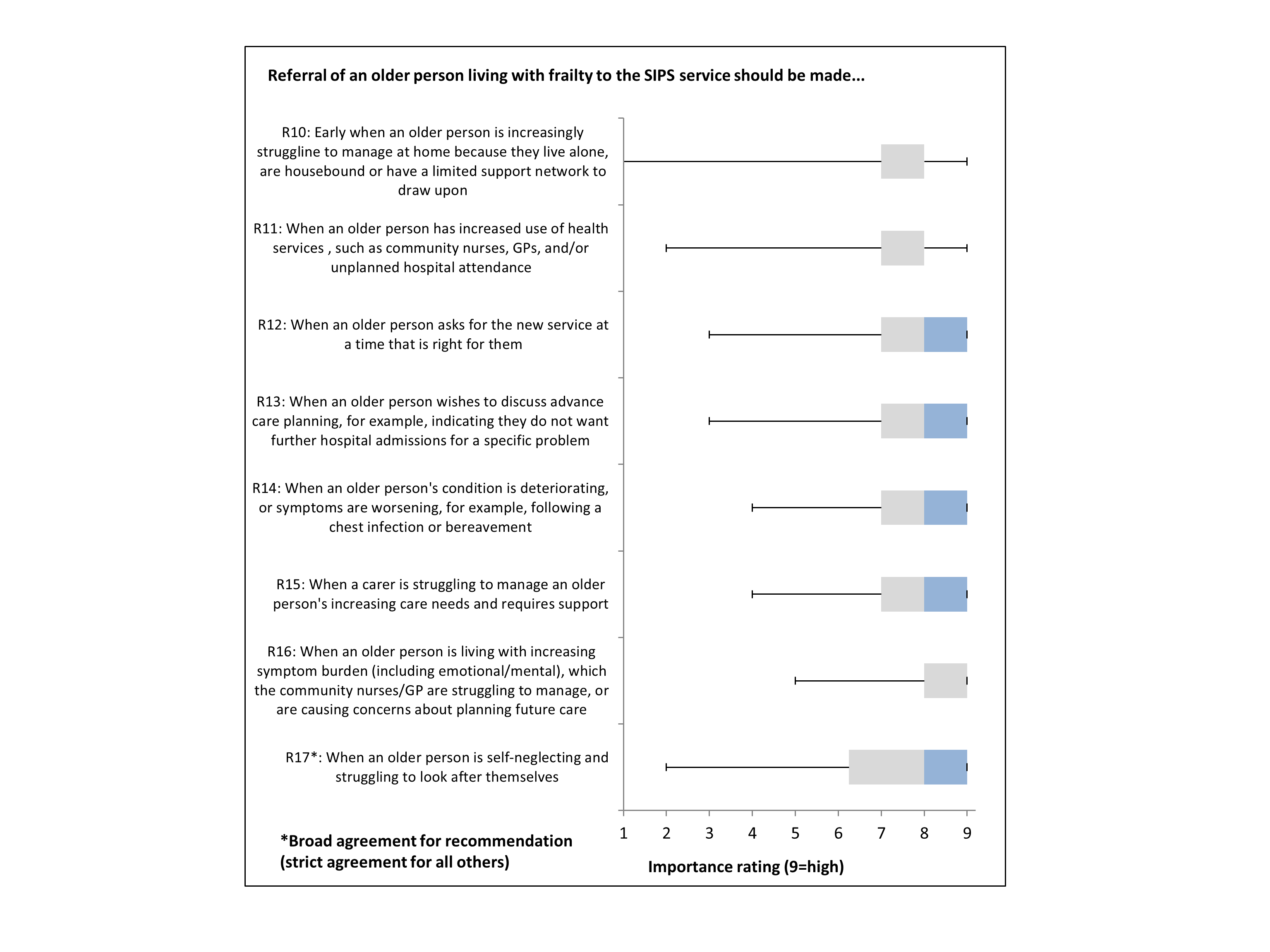
**

**Importance rating (9=high)**

***Broad agreement for recommendation (strict agreement for all others)**

**Suppl. Figure S4: Stakeholder recommendations for integrated working and consensus survey findings (mean, range and interquartile range**

**
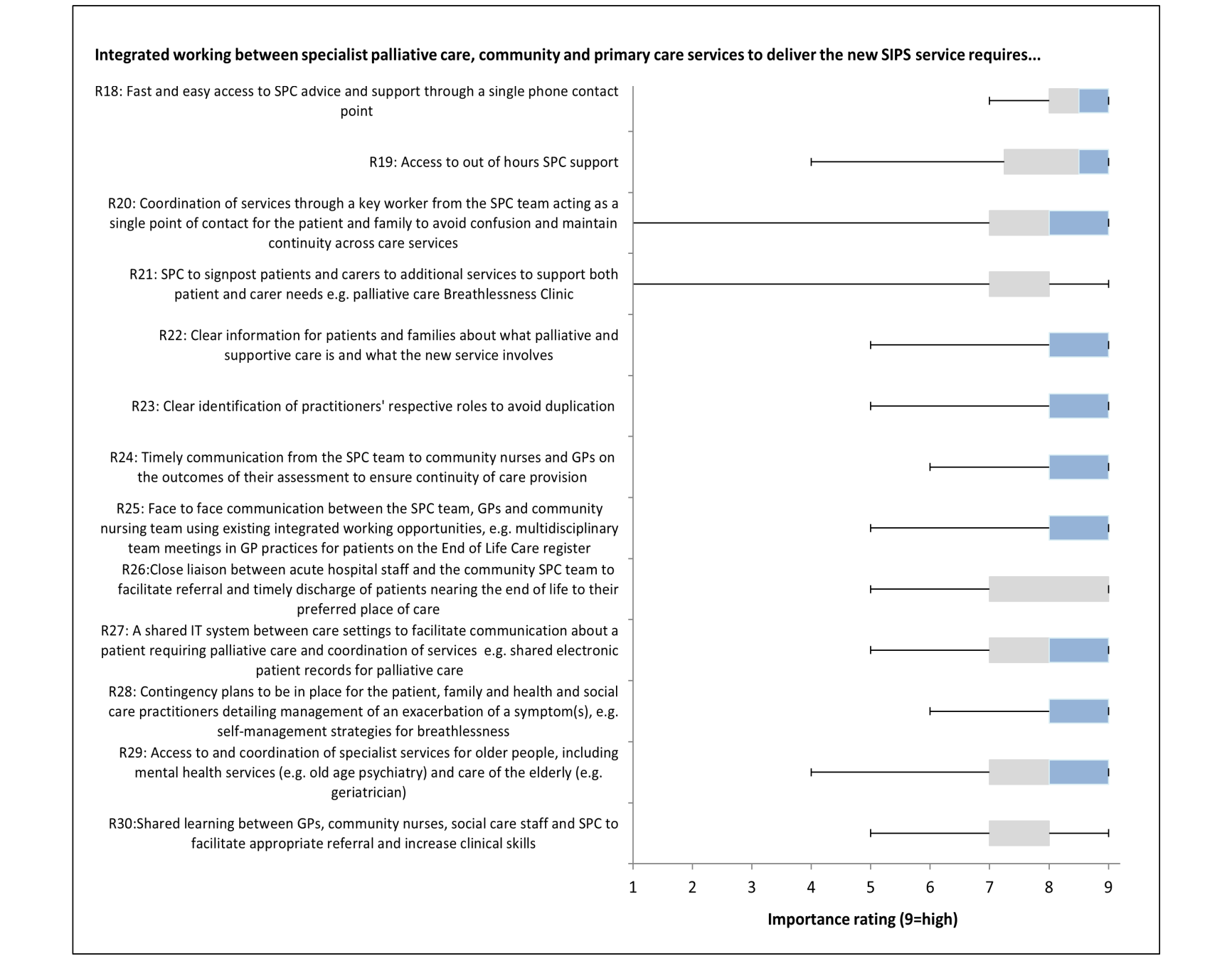
**
